# The “FitBrain” program: implementing exergaming & dual-task exercise programs in outpatient clinical settings

**DOI:** 10.3389/fspor.2024.1449699

**Published:** 2024-12-06

**Authors:** Ryan M. Glatt, Corwin Patis, Karen J. Miller, David A. Merrill, Brendon Stubbs, Manuela Adcock, Eleftheria Giannouli, Prabha Siddarth

**Affiliations:** ^1^ Pacific Neuroscience Institute and Foundation, Santa Monica, CA, United States; ^2^ Department of Psychiatry and Biobehavioral Sciences, David Geffen School of Medicine at the University of California Los Angeles, Los Angeles, CA, United States; ^3^ Department of Translational Neuroscience, St. John's Cancer Institute, Santa Monica, CA, United States; ^4^ Institute of Psychiatry, Psychology, and Neuroscience, King’s College London, London, United Kingdom; ^5^ Department of Sport and Human Movement Science, Centre for Sport Science and University Sports, University of Vienna, Wien, Austria; ^6^ Department of Research, Dividat AG, Schindellegi, Switzerland; ^7^ Department of Health Sciences and Technology, ETH Zurich, Zurich, Switzerland

**Keywords:** dual-task, exergaming, cognitive-motor dual-tasking, cognitive-motor training, neurological, older adults, rehabilitation, serious games

## Abstract

Dual-task training and exergaming interventions are increasingly recognized for their potential to enhance cognitive, physical, and mood outcomes among older adults and individuals with neurological conditions. Despite this, clinical and community programs that use these interventions are limited in availability. This paper presents the “FitBrain” program, an outpatient clinical model that combines dual-task and exergaming interventions to promote cognitive and physical health. We review the scientific rationale supporting these methods, detail the structure and methodology of the FitBrain program, and provide examples of session designs that integrate dual-tasking through exergaming. The paper also addresses implementation considerations, such as tailoring interventions to specific populations, ensuring user-centered design, and leveraging accessible technologies. We discuss key challenges, including limited research on programs utilizing multiple technologies and cost constraints, and propose directions for future research to refine best practices and evaluate the comparative effectiveness of multimodal vs. singular interventions. This paper aims to inform clinicians and program developers on implementing dual-task and exergaming interventions within diverse clinical and community settings by offering a structured model and practical guidelines.

## 1 Introduction

With the aging global population, cognitive impairments and neurological conditions are becoming increasingly common, imposing significant burdens on individuals and healthcare systems. Millions of older adults live with conditions such as Mild Cognitive Impairment (MCI) and dementia, both of which incur substantial personal and societal costs (1–5). Non-pharmacological strategies, especially physical activity and exercise, have shown potential in addressing this challenge by mitigating cognitive decline and enhancing physical function in older adults (1–7). Exercise interventions have shown benefits for physical and cognitive functions, even in individuals with cognitive impairments (4–6).

Exergaming and dual-task training appear to support cognitive and physical health through multiple mechanisms. These interventions promote neuroplasticity (8, 9), particularly in the prefrontal cortex (10), enhancing executive function and gait performance (11, 12). They also improve attention-shifting abilities and cognitive load management, helping to maintain balance and reduce fall risk (13–15). Dual-task training increases cognitive efficiency and information processing speed (16), benefiting overall cognitive performance, particularly in individuals with lower baseline cognitive function (8, 9, 17–22).

### 1.1 Exercise and cognition

In addition to exercise, cognitive training has emerged as another non-pharmacological intervention to enhance cognitive functions. However, the evidence supporting the efficacy of cognitive training alone remains inconclusive, with mixed results reported across numerous studies (7, 23, 24). Recognizing the potentially limited effects of either exercise or cognitive training alone, there has been growing interest in combining these interventions. Simultaneous cognitive and physical training may better mimic real-life demands and is more effective in improving cognitive outcomes than sequential training (16). Herold et al. (17) suggest that cognitive tasks should be integrated into motor tasks rather than treated as separate entities for optimal improvement in cognitive and motor functions.

### 1.2 Simultaneous cognitive and physical training approaches

The meta-analysis by Zhu et al. (19) found that interventions combining cognitive and physical exercise provide superior cognitive improvement compared to physical exercise alone in healthy older adults. These cognitive gains are observed over various periods, but the meta-analysis particularly highlights sustained effects for durations extending up to 12 months. This suggests that combined interventions are effective for short-term and long-term improvement of cognitive functions. Two approaches to incorporating simultaneous cognitive and physical training include dual-tasking and exergaming. Dual-tasking refers to the simultaneous performance of a cognitive and physical task, designed to mimic real-world multitasking situations. Exergaming, on the other hand, involves engaging in physical activity through interactive video games that require physical movement to control the game. While both approaches aim to improve cognitive and physical outcomes, dual-tasking focuses on integrating two distinct tasks, whereas exergaming uses gamification to motivate physical activity. However, exergaming incorporates elements of dual-tasking, and therefore exergaming could also be considered a dual-tasking intervention, with the addition of visual gamification elements. Both exergaming and dual-task training interventions have been studied for their role in the rehabilitation exercise and cognitive training in multiple clinical populations, including in older adults with and without cognitive impairments and in neurological populations such as those with Parkinson's Disease and Multiple Sclerosis (8, 9, 19–23).

### 1.3 Implementation of dual-tasking and exergame training

Current research has identified modest yet significant improvements in dual-task abilities resulting from simultaneous cognitive and physical training compared to separate implementations (19). This is particularly evident in older adults (16, 19), individuals with MCI and dementia (8, 9), and those with neurological conditions such as Parkinson's Disease and Multiple Sclerosis (25–27). The ability to dual-task or perform two separate tasks simultaneously tends to decline with age and the onset of neurological conditions, correlating with overall cognitive decline and increased fall risk (21, 22). Despite the promising evidence, the clinical implementation of dual-task and exergaming interventions remains limited. There is a need for structured programs that integrate these interventions within clinical settings to enhance their accessibility and feasibility. A structured methodological program could address these barriers by providing clear protocols, training requirements, and operational considerations, thus facilitating the integration and scaling of these interventions in clinical practice.

### 1.4 Theoretical approach of a “brain gym program”

The “brain gym program” proposed here draws on established cognitive rehabilitation models, enhancing them by combining cognitive-motor training with dual-tasking interventions in clinical settings. The brain gym program proposes a comprehensive and evidence-based approach by integrating multiple exergaming and dual-task interventions into a cohesive program. This model is akin to the “Clinical Arcade” concept in cardiac rehabilitation, which utilizes a variety of exergames to target specific rehabilitation goals (28). Integrating multiple modalities in the brain gym maximizes therapeutic benefits and enhances patient engagement and adherence by offering a diverse range of cognitive and physical challenges. Research has shown that such multimodal strategies can significantly improve cognitive functions, physical health, and dual-task abilities, providing a robust framework for addressing the complex needs of older adults with neurological conditions (12, 19, 29, 30).

## 2 Literature review

In conducting a review for this manuscript, we employed an approach to ensure a comprehensive and up-to-date analysis of dual-tasking and exergaming interventions aimed at improving cognitive and physical outcomes in older adults. We searched multiple academic databases, including PubMed, Scopus, Web of Science, and PsycINFO, using keywords such as “dual-tasking,” “exergaming,” “cognitive training,” “physical exercise,” “older adults,” and “neurorehabilitation.” The search was limited to studies published in English within the last 10 years, though seminal works outside this period were considered if frequently cited up until June 15th, 2024. This manuscript did not adhere to the full methodological requirements of a systematic review, nor did it conduct a formal data extraction or assessment of bias.

### 2.1 Review of exergaming interventions

“Exergames” are active (video) games that require physical interaction in response to visual or auditory stimuli (31). These games fall under the Games for Health Taxonomy and are primarily classified as “preventative” interventions, with their therapeutic use in conditions like dementia referred to as “Rehabilitainment” (32). Exergaming is a multi-faceted training approach that addresses various elements of exercise sciences (with acute variables such as dosage, duration, intensity, and frequency), targeted domains (cognition, physical function), populations (older adults, cognitive impairment, neurological conditions), and settings (community, clinical, or research) (33).

Exergaming examples include the Nintendo Wii, Microsoft Kinect system, stepping and dance games like Dance Dance Revolution, exergaming treadmills, exergaming cycling, and camera-based (i.e., Kinect, webcam) games (11). These can be general, i.e., not targeting specific cognitive or physical outcomes [e.g., Wii Sports (34)], or specific, aimed at outcomes (12). Specific exergames, termed “clinical exergames” in clinical settings, target rehabilitation goals, with some settings adopting a “Clinical Arcade” concept (28). Examples of specific exergames with clinical utility include CyberCycle (35), Dividat Senso (25), and SMARTFit (36), which are further discussed in Section 3.1. While specific exergames have not been shown to produce superior effects to general exergames, the therapeutic target of specific exergames is theorized to be more targeted (12, 34). Treadmill walking is considered an exergame when synchronized with interactive visual or auditory stimuli that require user response.

Randomized controlled trials (RCTs) indicate that exergaming may prevent and alleviate age-related cognitive decline in healthy older adults and individuals with neurological conditions (25, 26), including in those with cognitive impairments (37, 38). Exergaming has been found to improve neuropsychological functions comparably to other physical activities in healthy older adults (39). A meta-analysis of 17 RCTs showed significant improvements in executive functioning (g = 0.256, *p* = 0.048), attention (g = 0.298, *p* = 0.027), and visuospatial skills (g = 0.345, *p* = 0.033) with a moderate effect size on global cognition (g = 0.436, *p* = 0.001) through exergaming in clinical and non-clinical populations (11). Exergames are perceived as less intense yet maintain similar physiological intensity as traditional exercises, making them suitable for those hesitant to engage in conventional exercise methods (40). Currently, the authors have not identified any particular type of exergame that demonstrates superior effects on cognitive function, as head-to-head comparisons of exergame types have not yet been conducted.

### 2.2 Review of dual-tasking interventions

Dual-task techniques require participants to engage in cognitive processes (e.g., memory recall and problem-solving) while performing physical actions (e.g., walking, balancing). These techniques are designed to mimic real-life scenarios that require allocation towards more than one task, thereby simultaneously demanding cognitive and physical functions. Dual-tasking requires performing two independent tasks simultaneously, each with distinct goals (41). As McIsaac et al. (42) describe, dual-tasking differs and can involve dual cognitive-motor or motor-motor tasks, with complexity and novelty varying based on the intervention's goals (41). Dual-task interventions are used in diverse populations, including those with neurological conditions, to improve cognitive and physical abilities and transfer competencies to everyday life dual-task demands in everyday life. The specific cognitive and physical tasks involved vary considerably. Fritz et al.'s systematic review (21) highlighted the effectiveness of motor-cognitive dual-task training in enhancing gait velocity, stride length, balance, and cognition in Parkinson's and Alzheimer's patients (21). This review (21) covered 14 studies on dual-tasking, with interventions involving verbal fluency and naming tasks, serial subtractions (i.e., −3's and −7's), information processing speed tasks, counting tasks, ball-catching and hand-eye coordination tasks, and responding to questions while conducting gait or balance tasks. However, no analyses have been of what techniques were more or less effective than others (27, 42–45).

Other dual-task interventions include balancing while engaging in ball activities (13), Dual-task Zumba Gold (46), stepping and squatting with verbal tasks (47), square-stepping exercises (48), and navigating obstacles while counting or distinguishing sound tones (14). Studies by Weightman et al. (49) and Silsupadol et al. (14) identified various cognitive-motor dual-tasks targeting older adults with balance impairments, encompassing diverse secondary cognitive tasks alongside primary physical tasks, as detailed in Table 1.

**Table 1:** Examples of domain-specific cognitive tasks for cognitive-motor dual-tasks from weightman et al. (49) and Silsupadol et al. (14) with Targeted Cognitive Domains.

Task name(s)	Example(s) of domain-specific tasks	Targeted cognitive domain(s)
Auditory discrimination	Identify and name noises (cat vs. dog), voices (man vs. woman), or sounds (high vs. low tones)	Auditory Processing Speed, Selective Attention
Naming	Animals, states, foods, flowers, names of people	Verbal Fluency, Information Processing Speed
Visual Discrimination	“Spot-the-difference” tasks (does the picture shown matches the picture seen before; yes or no?)	Visuospatial Working Memory
Random Digit Generation	Randomly name numbers between 0 and 300	Information Processing Sped
Backward Counting	Counting backward by 2's, 3's, or 7's	Attention, Working Memory
Visuospatial	Place or name a number, object, or letter in a real or imagined space; recite or recall directions from or to a specific location.	Visuospatial Working Memory
N-Back	Recite numbers, days, or months backward or skip every other target in the sequence.	Working Memory
Subtracting Numbers from Letters	Provide a letter as the result of an equation (k-1 = j)	Working Memory
Memorization and Recall	Memorizing or recalling objects, names, words, numbers, or prices	Short-Term Visual or Spatial Memory
Story Telling/Recall	Tell a story about a day or vacation or recall details of a told story.	Episodic Memory
Opposing Directions	State or move in the opposite direction of action (if a left leg is moved, say, or move to the “right,” or if a ball is kicked to the right, say, or move to “left”).	Inhibitory Control, Attention
Backwards or Forwards Spelling	Spelling a word, or series of words, forwards or backward. Skipping letters in forward or reverse order.	Working Memory
Sentence Generation	Stating a complete a sentence, completing a sentence or idiom	Verbal Fluency, Attention
Stroop	Naming the color of the ink but not the word (if the word pink is colored in the ink “blue,” say “blue”)	Information Processing Speed, Inhibitory Control

### 2.3 Comparing exergaming to dual-tasking

Dual-task interventions, which require individuals to engage in both cognitive and physical tasks simultaneously, have shown efficacy in enhancing cognitive and physical functions across various groups, including healthy older adults (29), individuals with neurological conditions such as dementia and Parkinson's Disease (9, 21), and those with MCI (30). Exergames, a subset of dual-task interventions, often incorporate cognitive-motor dual-tasking, where users perform physical actions in response to cognitive challenges. General-purpose exergames, such as those on the Nintendo Wii, provide nonspecific cognitive stimulation but still improve cognitive and physical function (34). In contrast, purpose-built exergames are specifically designed to target defined cognitive domains and physical skills. As Wollensen et al. (12) highlighted, general exergames engage broad cognitive functions, whereas specific exergames are structured to align closely with targeted cognitive domains that match the outcome measures collected. This distinction underscores how general and targeted dual-task interventions can be applied through various modalities, including exergaming, to optimize specific functional outcomes.

It is notable that many exergaming devices primarily deliver cognitive stimuli. To constitute a dual-task paradigm, users must engage in concurrent physical activity. However, some exergames, offering visual stimuli requiring physical responses, may intermittently halt movement, which McIsaac et al. (42) might classify as a “complex single task” (41), still offering potential benefits. Currently, there is a scarcity of accessible dual-task training programs and exergame technologies in community and clinical settings. Notably lacking are those addressing cognitive and physical health in older adults and individuals with neurological conditions, focusing on specific cognitive domains such as executive functions, processing speed, and memory (39, 50). To be effective in clinical and community settings, exergames and dual-tasking interventions must be population-specific, feasible, and engaging; for example, selecting specific techniques, games, or tasks based on condition (such as stroke or Parkinson's Disease), cognitive domain (such as memory or processing speed) and physical function (such as balance or cardiovascular endurance) (12, 21, 30, 39, 51, 52).

## 3 The “brain gym” program

The “brain gym” program is deeply rooted in cognitive rehabilitation theories and supported by research on multimodal exercise interventions. Recent studies demonstrate that combined cognitive and physical training is more effective in improving cognitive outcomes than either intervention alone (8, 9, 17, 19–22). Furthermore, dual-task and exergaming interventions, like those explored in the FitBrain program (which is the specific brand name of a “brain gym” at the Pacific Neuroscience Institute), have significantly enhanced cognitive and physical functions across diverse populations, including older adults with neurological conditions (23, 29, 30). By systematically integrating multiple exergaming and dual-task interventions within a unified framework, the brain gym program draws on these established protocols and extends them by offering a more comprehensive and adaptable approach tailored to the needs of older adults and individuals with cognitive impairments. Currently, no preliminary evidence demonstrates that participation in a program such as FitBrain improves cognitive or physical function.

### 3.1 Single-modality vs. multi-modality approaches

Several existing studies focus on single-modality interventions, such as one game, device, or analog dual-task techniques. The brain gym program proposes a more comprehensive approach by integrating multiple exergaming and dual-task interventions into a unified program. Similar to successful multimodal exercise interventions, this multimodal strategy has yielded superior outcomes in various domains (53, 54). For instance, the “Clinical Arcade” concept in cardiac rehabilitation utilizes a range of exergames tailored to specific rehabilitation goals, demonstrating the potential benefits of this integrated approach (28). A similar model, adapted for geriatric and neurologic populations under the “brain gym” framework, offers a comprehensive, targeted intervention that addresses these populations’ complex cognitive and physical needs. However, no specific data has been reported comparing single-modality vs. multi-modality approaches.

### 3.2 “FitBrain” program overview

**Figure 1:**
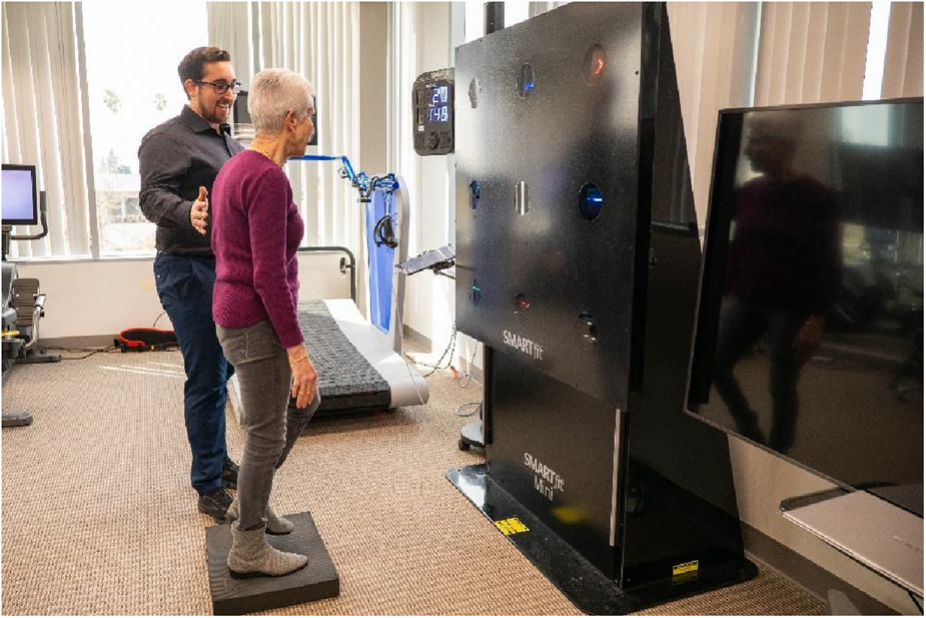
An image of the “FitBrain program” space, with the SMARTFit technology shown with a participant balancing on a foam pad while searching for two symbols that match.

The FitBrain program (Figure 1) is specifically designed to address older adults’ cognitive and physical needs, utilizing a combination of advanced technologies and evidence-based methods to deliver personalized interventions. The program occupies approximately 3,000 square feet, akin to a medium-sized physical therapy clinic or fitness space (in the USA), featuring areas for group activities, functional training, and individual treatment rooms shared by physical therapists and medical fitness professionals. The design prioritizes functional movement, with equipment arranged along the walls and ample open space for interaction and mobility tasks. The methodology section details the intervention protocols used in the FitBrain program, including the rationale for selecting specific exergames and dual-task tasks. Each protocol is designed to be adaptable to various clinical populations, with examples of session structures provided to illustrate how interventions can be customized based on individual needs.

### 3.3 Overview of dual-task training and exergaming technologies used

Dual-task training and exergaming interventions often leverage technology, such as screens, cameras, tablets, touch-sensitive targets, floor-based markers, and Virtual Reality (VR) headsets. While non-technological dual-tasks are feasible and already adopted in rehabilitation settings, more technology-dependent interventions may offer specificity, variability, complexity, engaging stimuli, and potential saliency. Fitness and rehabilitation centers often seek to integrate novel technology and approaches to diversify their programming. However, adopting evidence-based exergaming and dual-tasking interventions and technologies tailored to specific populations remains lacking. There are currently no studies looking into the potential combination of these interventional approaches in comparison with singular interventions (for example, the effectiveness of using a single exergaming technology vs. multiple exergaming technologies or the combination of technology-enabled dual-tasking interventions vs. non-technological dual-tasking interventions). However, the integration of numerous exergame and dual-task approaches may increase adherence through variety and enjoyment while allowing the targeting of multiple cognitive and physical functions.

The subsequent sections categorize the dual-task and exergaming technologies into “cardiovascular”, “reaction-based,” and “gaming-based.” While more categories exist, these categories summarize the currently utilized technologies within the FitBrain program based on their interface, training purposes, and implementation. Cardiovascular technologies typically involve stationary bikes or treadmills paired with visual stimuli delivered through screens, tablets, or VR headsets, complemented by dual-task techniques administered by professionals. “Reaction-based” technologies utilize screens, LED lights, and sensor-based systems requiring users to interact with specific targets. “Gaming-based” exergaming technologies engage users in various game paradigms, demanding physical responses to interact with a gaming environment or tasks. Given that all interventions may include aspects of “gamification” and “dual-tasking”, it is difficult to separate these interventions simply by either label, and there it should be assumed that all exergames include aspects of dual-tasking, and dual-tasking interventions may include one or more elements of gamification (e.g., score, competition, goals, etc.).

#### 3.3.1 Cardiovascular exergaming and dual-task technologies

**Figure 2:**
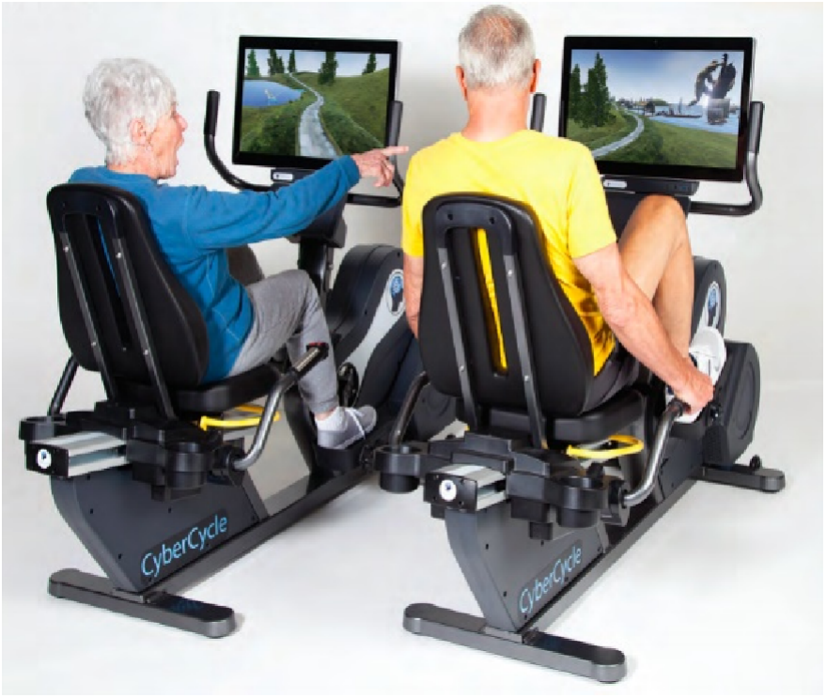
Cybercycle.

CyberCycle (Figure 2): The CyberCycle (Blue Goji, Austin, TX) is a recumbent exercise bike with an interactive screen and handlebars for steering an on-screen character through virtual environments. Earlier studies suggest that adherence to a 12-week CyberCycle program may lead to improvements in executive function and a modest risk reduction of MCI conversion (55–57).

**Figure 3:**
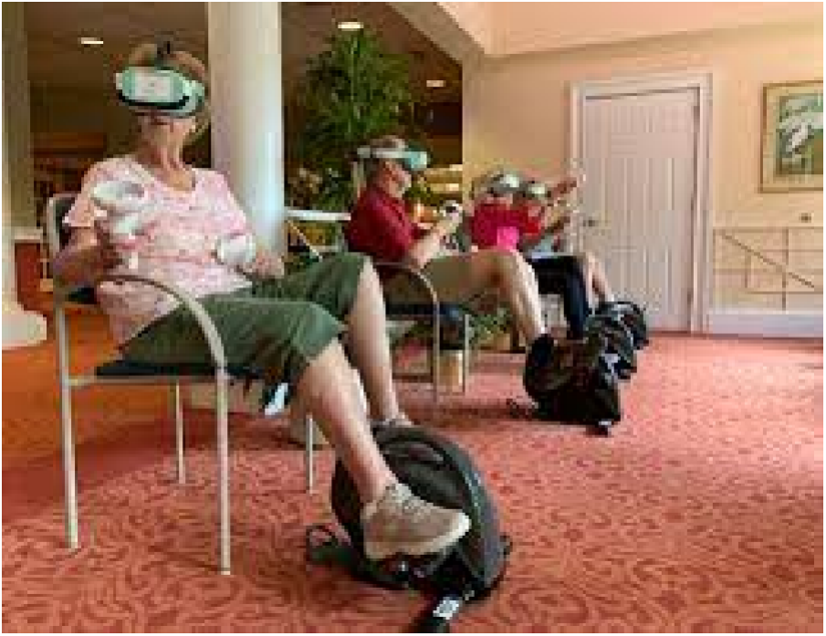
Rendeverfit.

RendeverFit (Figure 3): RendeverFit (Rendever, Somerville, MA) is an immersive VR platform that includes activities such as Cycle, Paddle, and Paint, merging physical and cognitive stimulation with social engagement for individuals with major neurocognitive disorders. While its effectiveness on physical and cognitive functioning is currently being studied, some initial evidence from Rendever's social aspects suggests it may reduce social isolation and enhance well-being in older adults (58, 59).

**Figure 4:**
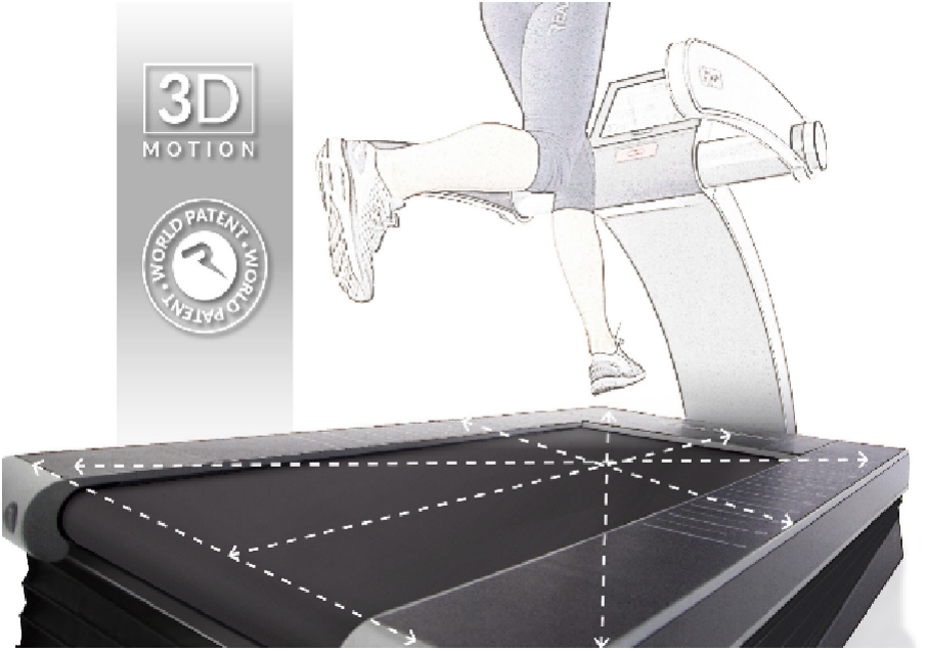
Reaxing treadmill.

ReAxing (Figure 4): ReAxing (Reaxing USA, Simi Valley, CA) uses tools like tilting treadmills and fluid-filled resistance implements to create unpredictable perturbations that increase neuromuscular demands. It aims to address fall risks in older adults and those with neurological conditions. Preliminary research suggests potential improvements in balance in older adults and neurological populations from perturbation training (such as slip-based perturbations, which do not use ReAxing equipment) (15, 60, 61). However, no research has been published utilizing ReAxing tools (which use tilt-based perturbations).

#### 3.3.2 Reaction-based exergaming and dual-task technologies

**Figure 5:**
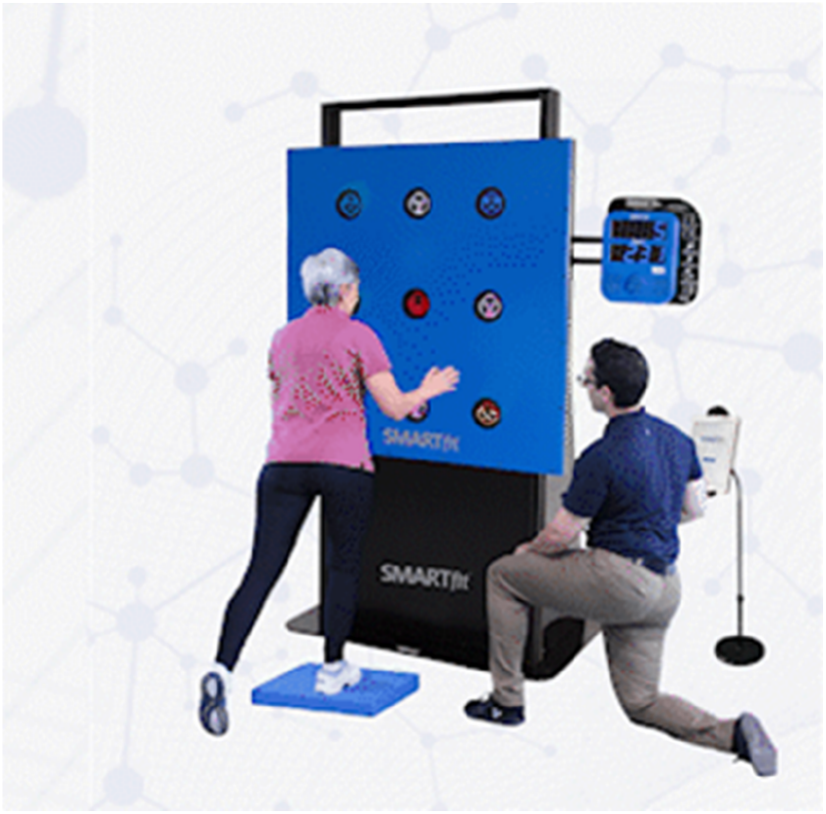
SMARTFit.

SMARTFit (Figure 5): SMARTFit (SMARTFit Inc., Camarillo, CA) features touch-sensitive LED targets controlled by a mobile app, combining cognitive tasks with physical exercises. Two recent feasibility studies in populations with Parkinson's Disease (62) and MCI (36) suggest that SMARTFit is safe, feasible, potentially productive, and enjoyable in clinical and community settings. Chua et al. (62) found that eight weeks of SMARTFit training may improve cognitive outcomes more effectively than standard physical therapy for Parkinson's patients. In addition, another study indicated significant improvements in balance and executive functions in older adults with MCI after 12 weeks of SMARTFit training (36).

**Figure 6:**
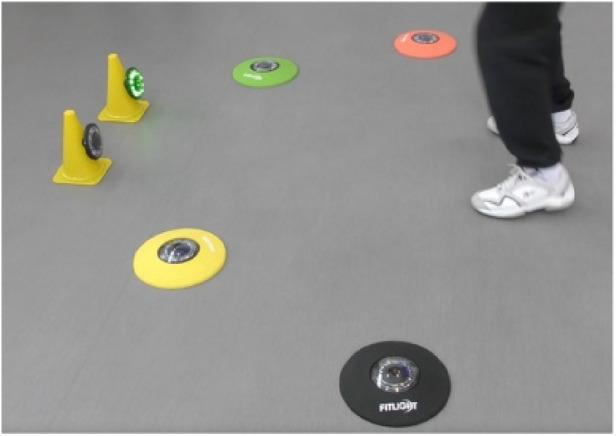
Fitlights.

FitLights (Figure 6): FitLights (Fitlight, Aurora, Ont., CA), a system of LED discs with touch and proximity sensors, can be used for agility, balance, and gait tasks and assessments. A pilot study found that 12-week FitLights training significantly reduced fall risk in older adults and provided valuable assessments of reaction time and anticipatory strategies in dynamic postural control dual tasks (63–67).

**Figure 7:**
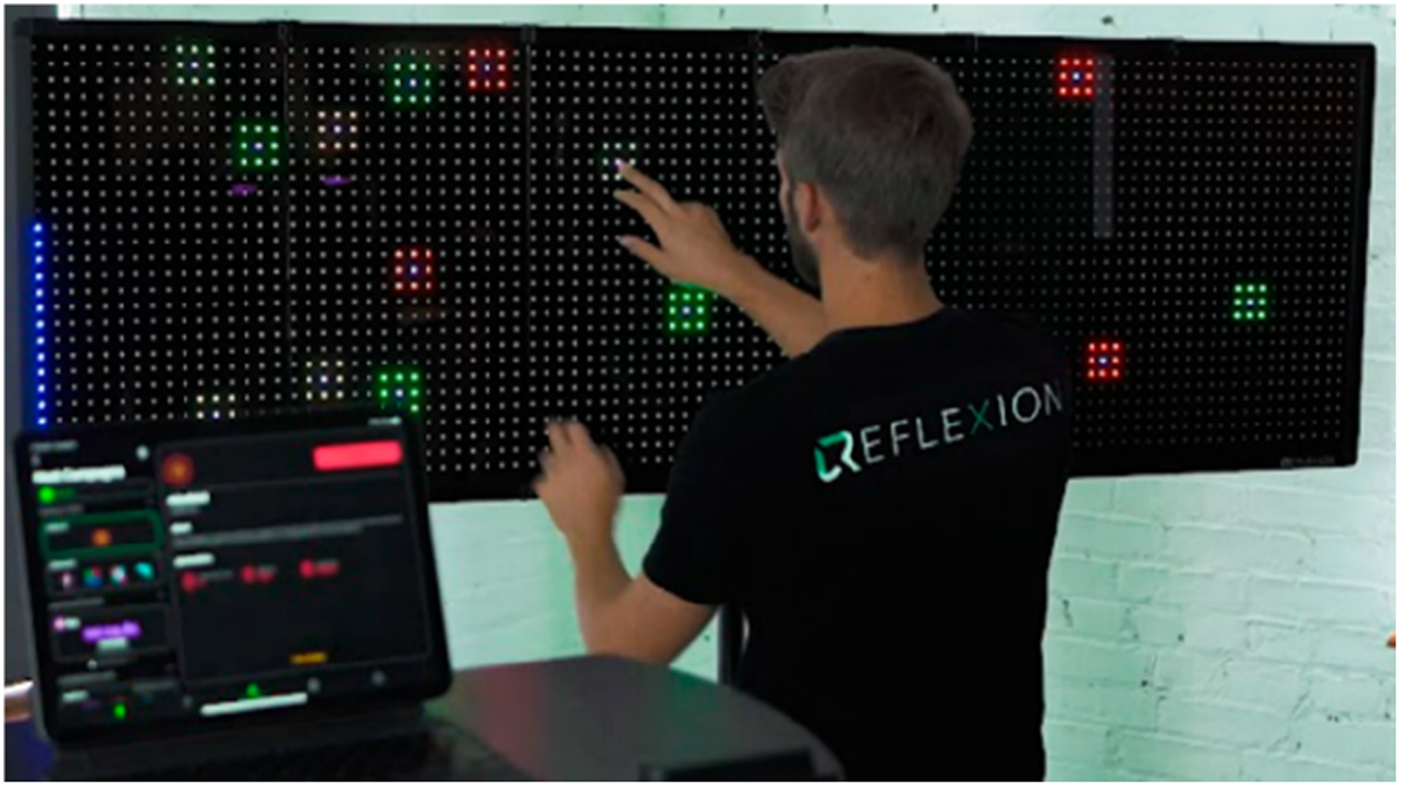
Reflexion.

Reflexion (Figure 7): Reflexion (Reflexion, Lancaster, PA), primarily developed for athletes, is an LED-based touch-screen board designed to train visual-cognitive skills. Although its initial research focused on athletes, there is potential for its application in older adults for visual-cognitive skill training, though further research is required (68).

**Figure 8:**
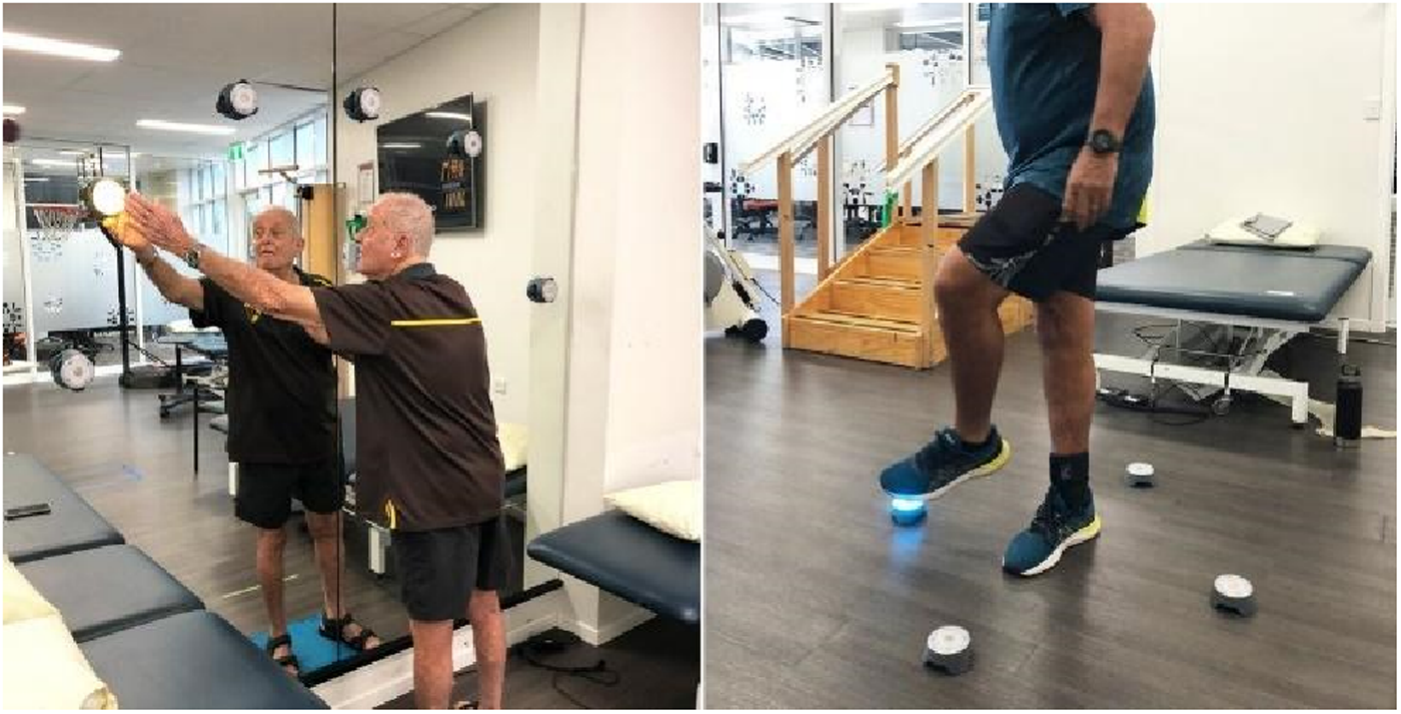
Blazepods.

BlazePods (Figure 8): BlazePods (BlazePod, Miami, FL) are modular, wireless lights controlled by a mobile app. They facilitate reaction-time tasks and are used in balance, agility, rehabilitation, and sport-specific exercises. Preliminary studies suggest BlazePods may be effective as a reactive assessment tool and improve these physical functions (69–76).

#### 3.3.3 Exergaming technologies with specific game paradigms

**Figure 9:**
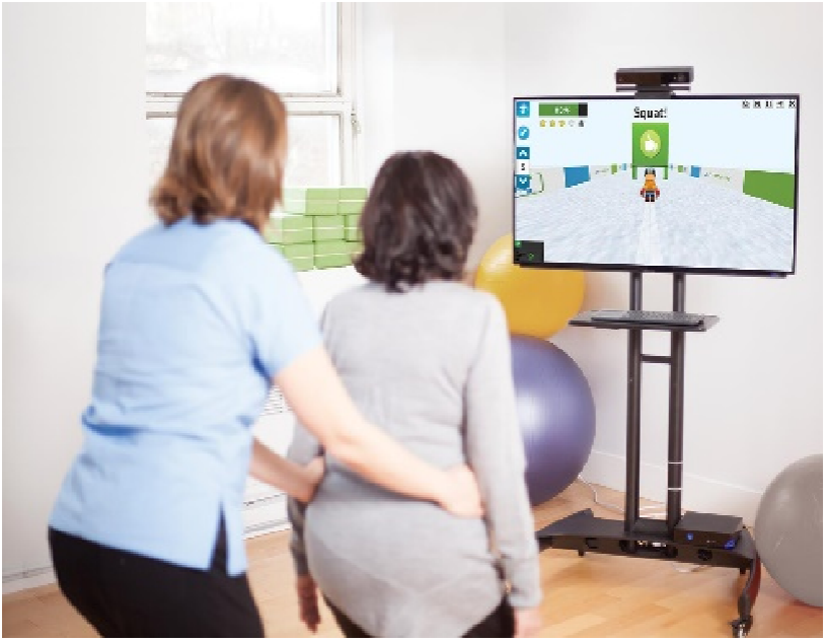
Jintronix.

Jintronix (Figure 9): Jintronix (Ludica Health, Montreal, QC, CA) utilizes Microsoft Kinect to offer games focusing on joint motion, dynamic movements, and cognitive exercises. Early studies have demonstrated safety, feasibility, and potential effectiveness in improving gait speed, physical functioning, and motor outcomes in various settings, including stroke rehabilitation and ICU environments (77–83).

**Figure 10:**
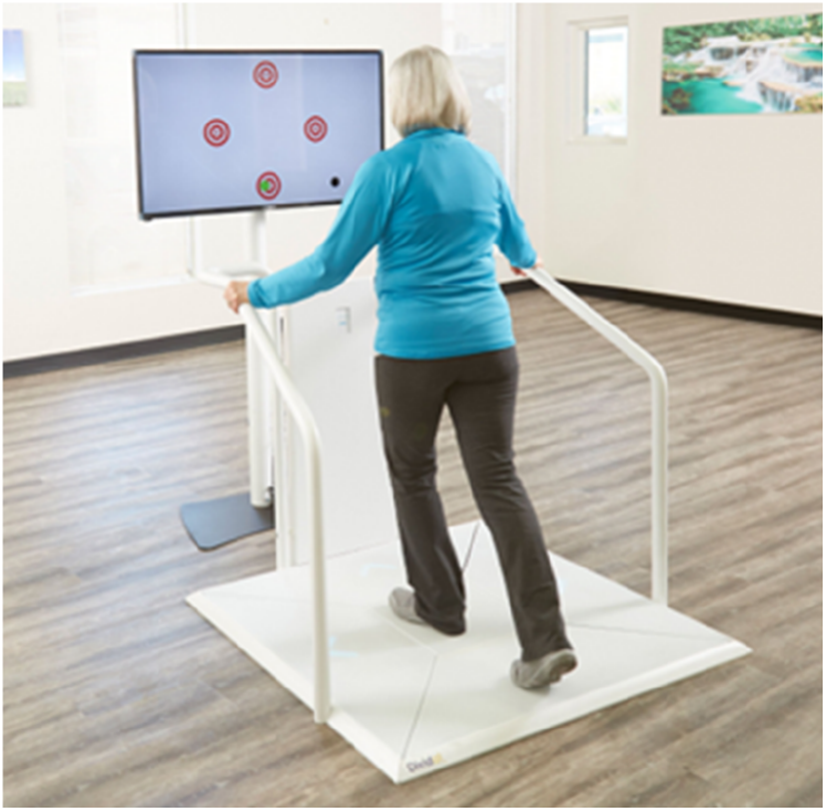
Dividat senso.

Dividat Senso (Figure 10): The Dividat Senso (Dividat AG, Schindellegi, CH) is a lower-extremity stepping-based clinical exergaming system featuring an LCD screen and floor-based arrangement of arrows activated by stepping and weight-shifting. Its exergames target physical functions such as balance, cardiovascular function, stepping strategies, and cognitive domains like attention, executive function, processing speed, and memory. Preliminary studies indicate it may be feasible and beneficial for individuals with conditions like Parkinson's Disease, multiple sclerosis, and vestibular hypofunction, as well as for older adults in rehabilitation settings (10, 84–89).

**Figure 11:**
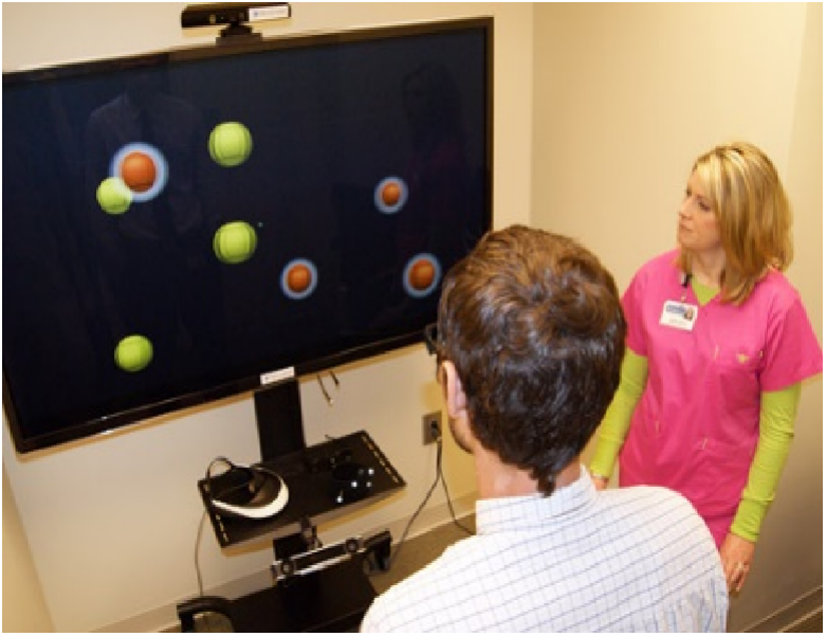
Neurotracker.

Neurotracker (Figure 11): Neurotracker (NeuroTracker, Montreal, QC, CA) is a 3D-MOT task paradigm software designed to enhance perceptual, cognitive, and visual abilities. The majority of Neurotracker's paradigms include tracking one or more targets at one time, ignoring distractor targets, for a set period of seconds, until being required to select the correct original targets. The task may be viewed in 2D, 3D, or virtual reality, with the number, color, and speed of targets being modifiable, in addition to the type of background. Some case studies suggest that its use may improve memory and processing speed in older adults, though more comprehensive studies are needed (90–94).

**Figure 12:**
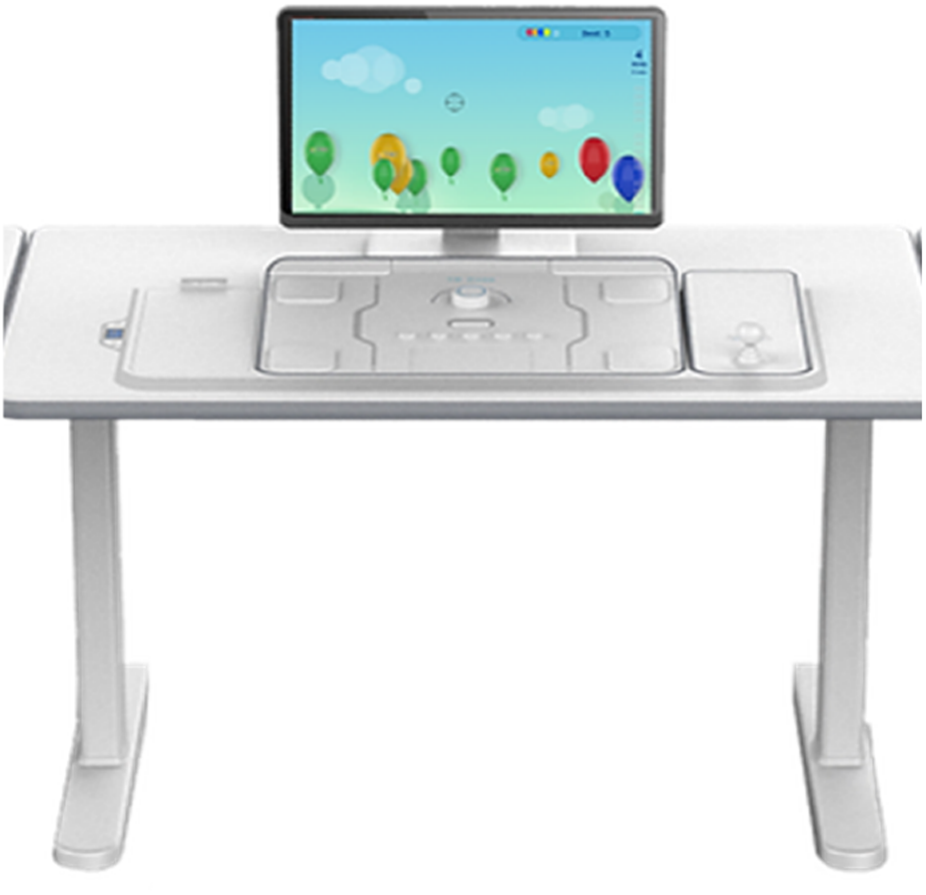
TRCare.

TRCare (Figure 12): TRCare's NeuroMotion system (TRCare Inc., Alviso, CA) is designed for post-stroke recovery, delivering therapeutic games to train specific physical tasks such as upper extremity function and fine motor skills through button-pressing mechanics with point-and-shoot games, card games (such as matching cards and blackjack) and games that involve pressing certain buttons at specific times with varying speeds. Early studies indicate it is feasible and effective in improving reaction time, coordination, and cognitive demands during early post-stroke recovery (95).

#### 3.3.4 Mobile, in-home & tele-rehab technologies

**Figure 13:**
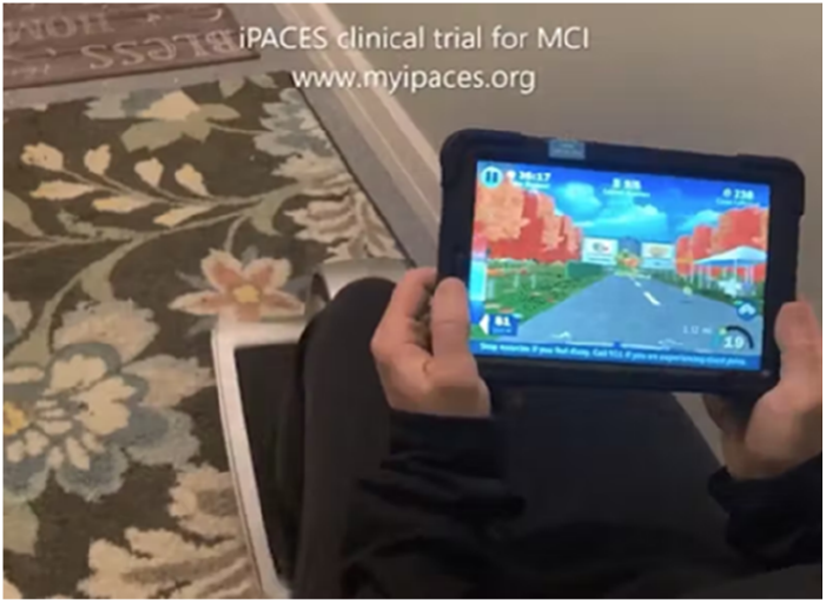
iPACES.

iPACES (iPACES, LLC, Clifton Park, NY) (Figure 13), a tablet-based exergame for exercise bikes, integrates cognitive tasks with physical exercise by asking users to select one of two “exits” by turning the tablet left or right (like a steering wheel) as a selection mechanism based on an initially presented list of words to be memorized in forward and then reverse order, showing promise in preliminary studies for improving executive function (96, 97).

**Figure 14:**
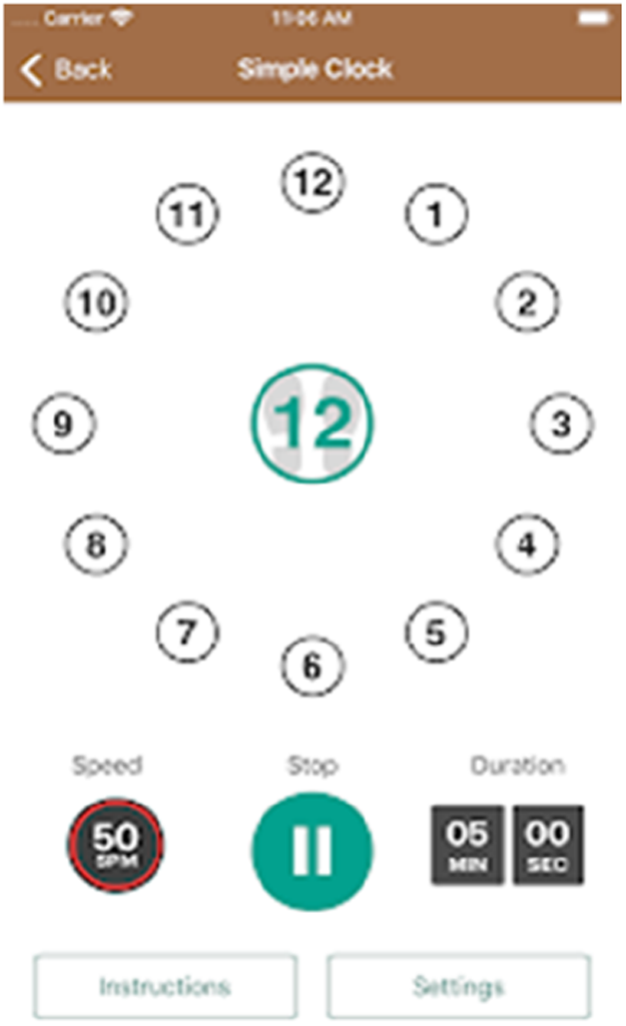
Clockyourself App.

**Figure 15:**
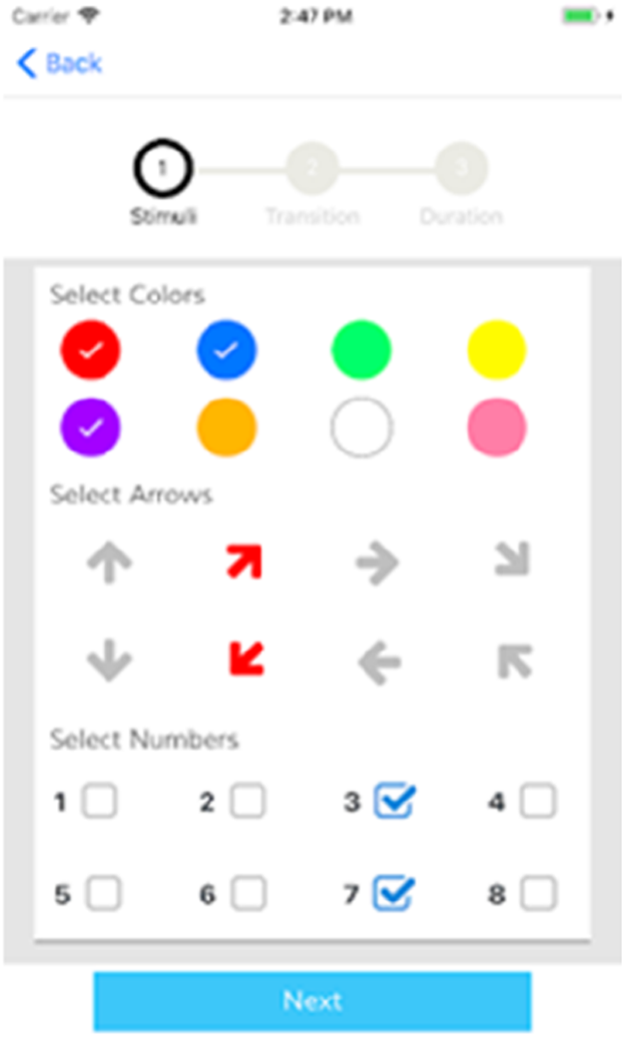
Switchedon training App.

Mobile apps, like ClockYourself (Next Step Health, QLD, AUS) (Figure 14) and SwitchedOn (SwitchedOn Training App, Chicago, IL) (Figure 15), combine physical and cognitive exercises, potentially aiding in fall prevention and cognitive function. However, further research is needed to solidify these findings (38, 96–101).

Virtual sessions within the FitBrain program are delivered through platforms like Zoom or Google Meet, offering both individual and group formats to support participants who may be unable to attend in person. These sessions are designed to extend the dual-task training environment into a home setting, making use of various techniques and tools to ensure effective engagement. Participants are expected to have basic technology access at home, including a computer or tablet with video conferencing capabilities.

In these virtual sessions, a variety of dual-task methods are employed, often incorporating auditory stimuli. For example, participants may use self-generated cues (such as counting backward or completing verbal fluency tasks listed in Table 1) or respond to real-time prompts from the instructor (such as stepping left upon hearing “blue” or performing a squat at even numbers). Visual aids, such as virtual drawing tools, are used to demonstrate movement patterns and create quadrants that participants can follow within their home space. Additionally, mobile apps like SwitchedOn (Figure 15) or ClockYourself (Figure 14) can be incorporated into these sessions via screen-sharing to enhance interactive participation, accommodating participants without specialized equipment.

**Figure 16:**
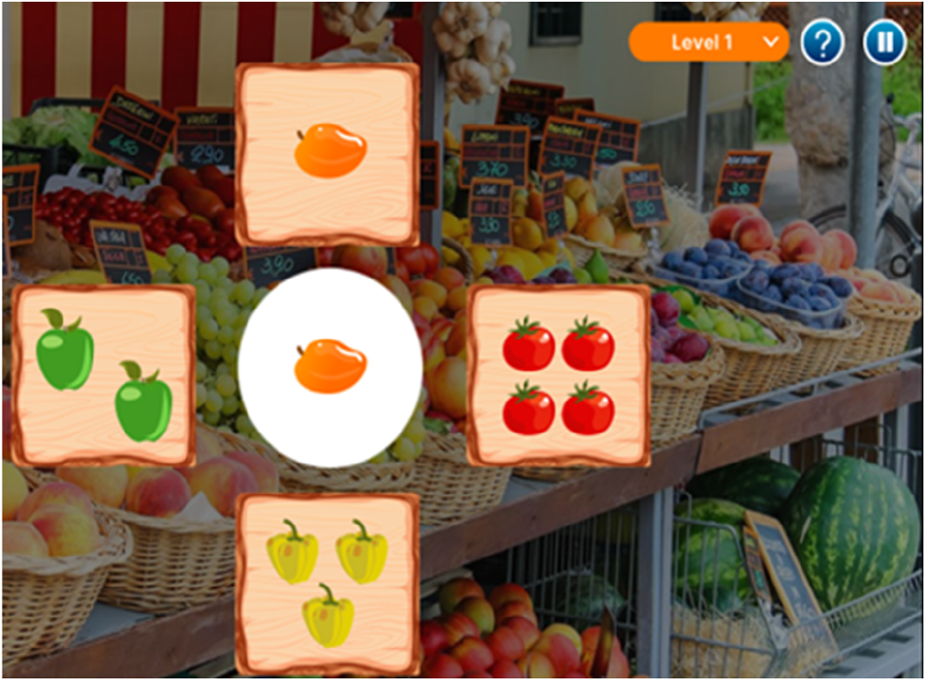
Virtual “FitBrain” games.

Custom computer programs or web-based tools may be used to design exercises where cognitive tasks cue physical movements, fostering a synchronized cognitive-motor experience. An example of this includes dual-task exercises developed in Unity on a web-app (“Virtual FitBrain Games”), illustrated in Figure 16, allows for structured dual-task exercises even in a virtual environment through teleconference screen-sharing, helping participants to benefit from the program from the convenience of their homes. These virtual sessions can be conducted with one individual or a group through teleconferencing tools.

## 4 Program inclusionary and exclusionary criteria

Participants are selected based on the following inclusion criteria: aged 50 years and older, cognitively healthy (as indicated by at least a 28/30 on the MoCA or equivalent), or diagnosed with MCI (as indicated by an 18–25 on the MoCA or equivalent) or early-to-moderate dementia (as indicated by a range of 10–17 or lower on the MoCA), neurological conditions (Parkinson's Disease, Multiple Sclerosis), acquired brain injuries (stroke, TBI), and capable of participating in physical activity as confirmed by a medical professional. Exclusion criteria included severe cognitive impairment (less than 10 points on the MoCA or equivalent or neuropsychological evaluation), significant physical limitations preventing exercise participation, and any severe neurological or psychiatric impairments that could interfere with the ability to engage in the intervention activities. For the FitBrain program, participants must be proficient in English, although some technologies offer multiple languages. Significant vision or hearing loss is included as an exclusion criterion due to potential limitations in using visual or auditory interactive technologies. However, this may be on a case-by-case basis depending on the types of techniques employed, as traditional non-technological dual-task techniques are primarily auditory, while the majority of exergames and screen or light-based technologies are visual.

### 4.1 Asesessments

The FitBrain program's intake process begins with a consultation and an initial assessment. Physical therapists conduct comprehensive evaluations using balance questionnaires (e.g., Activity-Specific Balance Confidence Scale) and physical activity assessments (e.g., Physical Activity Vital Signs). Tests include static balance (Four Stage Balance Test), dynamic balance (Four Square Step Test, which includes a change of direction and short-term memory of a series of steps, components present in dual-tasking), strength endurance (30 Second Sit-to-Stand, Grip Strength, 5× Sit-to-Stand), cardiovascular fitness (2 or 6 Minute Walk Test, 10 Meter Walk Test), mobility (Timed Up and Test), cognitive-motor dual-task ability (Timed Up and Go Cognitive Test), motor-motor dual-task ability (Timed Up and Test with an additional motor task), blood pressure, and sensory function (modified Clinical Test of Sensory Integration and Balance). Personal trainers can implement sub-clinical assessments, including those that are part of the Senior Fitness Test, which may provide some overlap with the assessments that rehabilitation clinicians can provide. Sessions introduce various exergames to evaluate difficulty, enjoyment, and relevance, with initial scores as baselines for future progress. “Less comprehensive” assessments refer to baseline functional fitness evaluations that do not integrate cognitive assessments.

Rehabilitation professionals may use additional measures before and after dual-task and exergaming interventions, such as the MMSE or MoCA assessments, pencil and paper tests, computerized cognitive tests, Quantitative Electro-Encephalography (QEEG), and volumetric or functional magnetic resonance imaging (vMRI, fMRI). Experimental measures might include dual-task balance tests like the Four-Square Step Test cognitive condition (which involves a serial subtraction task) (102) and variations of the Timed Up and Go Cognitive test with Oral Trail Making Parts A and B (103).

Plummer et al. (22) reviewed diverse dual-task gait assessments in older adults, such as counting exercises, verbal fluency tasks, and carrying objects, which include the Timed Up and Go Cognitive, Manual, and Triple conditions previously mentioned. Additional assessments include the Multiple Resource Questionnaire (104) and the Dual-Task Impact on Daily Living Questionnaire (105). Gait evaluation should cover single- and dual-task assessments and dual-task cost calculations. The Gait and Brain Study by Montero-Odasso et al. (106) showed that dual-task gait ability could indicate cognitive decline (107) and distinguish cognitive aging stages (108). In summary, despite the high number of potential outcome measures, practitioners select the outcome measures based on scope-of-practice, relevance to participant goals, evidence base, the type of participant (i.e., age, impairment, diagnosis), and allotted assessment time. However, a standardized assessment process may be developed to track outcomes across multiple participants.

### 4.2 Programming

The FitBrain program offers one-hour, one-on-one sessions with group and virtual options for cost-effectiveness. Sessions are tailored to individual goals and abilities and are implemented in a manner to overcome barriers for participation. The tailoring of sessions can be done via a decision tree, which includes a selection of specific tasks that address various cognitive domains, which are selected based on upon any objective or subjective cognitive assessments. Furthermore, physical assessment allows facilitators to customize the selection of physical tasks based on participant goals and limitations. Referrals come from various clinicians, and prospective participants get a program overview and a 30–60-minute consultation, which can be insurance-covered by a billable provider or paid out-of-pocket. Participants are provided with general education on exercise resources, including those in the community and digital resources, and information on general exercise guidelines. Participants typically attend one or two one-hour sessions weekly for 8 to 12 weeks, based on research on optimal exergaming and dual-tasking interventions for cognitive enhancement in older adults (11, 50).

The program intervention consists of 12-week sessions, with participants attending three 60-minute sessions per week. Each session includes a warm-up, followed by a series of dual-tasking and exergaming activities. Dual-task activities involve cognitive tasks such as memory recall or arithmetic performed simultaneously with physical tasks like walking or balance exercises. Exergaming activities are designed to target specific cognitive and physical domains, with progression managed by increasing the difficulty of cognitive tasks or the intensity of physical activities over time.

### 4.3 Intervention

The FitBrain program combines domain-specific cognitive exercises with targeted physical activities. For example, dual-task exercises to improve executive function and balance might challenge impulse control during a staggered stance. Sessions focus on cognitive domains such as executive function, attention, processing speed, and memory, often incorporating multi-domain interventions, as research suggests these may be more effective than single-domain training for improving cognitive outcomes like memory (109–111). Participants may engage in the program for 8–12 weeks, 1–2 times per week, and ongoing at a rate of 1–2 times per week as desired by the participant and program capacity.

The FitBrain program integrates exergame and dual-task technologies with neuromotor tasks, using equipment such as agility dots, cones, hurdles, balls, and various sporting gear. This program follows a structured approach, aligning with evidence-based guidelines, including the FIIT (Frequency, Intensity, Time, and Type) principles from the American College of Sports Medicine (ACSM) and the World Health Organization (WHO) recommendations for physical activity and exercise in older adults. Moderate-intensity exercise is encouraged and implemented, given the evidence that it can enhance cognitive function in older adults with and without cognitive impairments (1–7). The program supports comprehensive physical conditioning and includes aerobic steps, balance pads, and resistance tools like dumbbells, weighted bars, sandbags, and exercise bands. Sessions incorporate aerobic, resistance, and neuromotor exercises alongside cognitive-motor dual-tasking to promote overall health benefits.

The FitBrain program also uses analog methods for dual-task interventions, providing options for an affordable and low-tech approach. This includes non-electronic equipment like speed, agility, and quickness (SAQ) tools (hurdles, agility dots), balance implements (foam pads), agility ladders, marked cones, and mats with stimuli (colors, numbers, symbols, letters), as well as various projectiles (balls, balloons, cloth). These items are used in dual-task drills for physical tasks (balancing, stepping, navigating obstacles) or cognitive tasks (target identification, matching). Stimuli can be verbal or visual, incorporating cognitive tasks described by Weightman et al. (14) and Silsupadol et al. (49) (Table 1).

To ensure safety and effectiveness, the program uses a self-reported Rate of Perceived Exertion (RPE) to monitor physical exertion and assess the cognitive load, enabling tailored intensity adjustments based on each participant's physical and cognitive abilities. Techniques are adapted to individual goals and exercise tolerance, emphasizing balance and coordination for vulnerable populations. This approach provides a structured yet flexible training environment, modifying complexity and intensity to challenge participants’ physical and cognitive capacities safely. Table 2 shows an example of a FitBrain session based on available equipment. The results of the intake assessments help guide the programming and intervention selection, allowing for an individualized approach. Rest periods are not shown in the table, but 2-minute rest periods are provided in between exercises or as needed by the participant, with the duration of the task including a “practice round” of each task as needed based on the novelty of the task and prior experience and cognitive level of the participant.

**Table 2:** Sample dual-task and exergaming session structure.

Equipment	Technique/Task(s)	Targeted cognitive domains	Target physical domains	Duration
CyberCycle	Collecting Coins	Visuospatial, Attention	Cardiovascular	8 min
SMARTFit	Matching Like Symbols while Marching in Place	Complex Attention, Working Memory	Cardiovascular	8 min
Dividat Senso	Steps on one of four targets at the right time at varying speeds	Task-switching, Reaction Time	Dynamic Balance	8 min
Jintronix	Skiing Downhill with Weight Shifting and Squatting	Attention, Inhibition	Strength	8 min
SMARTFit	Selecting the Correct Sequence of Numbers while Hurdle Stepping	Working Memory, Set-Shifting	Dynamic Balance	8 min
Resistance Training	Counting Backwards While Conducting Weighted Moves	Executive Functioning	Neuromuscular	8 min

### 4.4 Program considerations for a “brain gym”

Comprehensive planning is essential when establishing a “brain gym” like the FitBrain program. This involves selecting appropriate equipment, tools, and software for exergaming and dual-tasking interventions, along with careful consideration of staffing, spatial requirements, assessment, and intake processes (which vary depending upon the practitioner and setting), implementation strategies for sessions (including the use of technology, virtual platforms, or traditional methods), business models, and tailoring the program to the intended demographic. The primary referrals are received from local neurologists and primary care providers, with at least 80% of referred participants completing at least 12 weeks of training. However, specific adherence and dropout rates are not reported in this manuscript. In terms of safety, no adverse effects have been observed or reported. The majority of participants indicate overall enjoyment in participation of the program.

Barriers to attendance can include cost of paying out-of-pocket if physical therapy services are not available, and transportation can be an additional challenge for some participants. There is an educational component of teaching providers how to utilize best and troubleshoot exergaming technology and disseminate best practices based on current literature (23, 52). In addition, general health education on healthy, evidence-based lifestyle behaviors such as exercise, nutrition, sleep, cognitive stimulation, and stress (18) are provided based on the request and willingness of participants. The costs associated with starting a program, including equipment, are an important consideration. While all the equipment overviewed in section 3.2 is part of the FitBrain program, it is unnecessary to possess all this equipment to run a program. Early iterations of the program included reaction lights (i.e., Fitlights, Blazepods), apps, agility equipment, and traditional dual-task techniques. Subsequent iterations included adding larger equipment (i.e., SMARTFit, CyberCycle), with the latest iterations including the equipment listed in section 3.2.

### 4.5 Staffing considerations

FitBrain's staffing model includes diverse healthcare professionals experienced with older adults and specialized populations. The team typically features board-certified physical therapists, ideally with Geriatric Clinical Specialist or Neurological Clinical Specialist designations, and personal trainers with an NCCA (National Commission for Certifying Agencies) accredited certification and specialized training in older adults, medical fitness and additional specialization in working with special populations. The program may incorporate occupational therapists, speech-language pathologists, neuropsychologists, and recreational therapists. Service sessions focus on movement and exercise, are offered in 30- or 60-minute segments, and can be individual or group sessions based on participant preferences and needs.

#### 4.5.1 Billing considerations

Billing practices depend on the provider's credentials. Services by qualified physical therapists may be eligible for insurance billing, while other services might require out-of-pocket payments, either as single sessions or packages. Insurance-billed services provided by Physical Therapists incorporate specific technologies and dual-task techniques into care plans to address the unique needs of patients, who may be referred for conditions like MCI, Parkinson's Disease, MS, or general gait and balance issues. The technologies and techniques used in the FitBrain program are selected to meet these individual deficits and needs.

## 5 Discussion

Exergames are well-received and feasible across various populations, including older adults (112). However, employing user-centered design practices (113) and customizing programs to the target population is essential to overcome barriers to adoption and participation (114). Given the visual nature of information delivery in exergames, considerations for individuals with visual deficits are essential, potentially necessitating a greater reliance on auditory stimuli. Accommodating a range of sensory abilities is vital, especially in older and neurologically diverse populations. Older adults with cognitive deficits often prefer exergames as they address both cognitive and motor functions effectively and enjoyably (23). Tailoring interventions to users’ specific cognitive, physical, and mood profiles may enhance cognitive functioning (39). Additionally, dual-task interventions should embrace the principles of progressive overload, incrementally increasing difficulty, complexity, intensity, duration, and task specificity to ensure transferability to daily activities (12).

### 5.1 Limitations of methods

This program's primary limitation lies in the lack of feasibility and effectiveness data and high equipment costs. These factors limit program scalability and affordability across diverse settings. Dual-task interventions exhibit considerable variation, as do the outcome measures used in research, particularly in neurological populations, posing challenges in comparison and highlighting the need for more robust randomized controlled trials (RCTs) (21). A notable issue is that many dual-task and exergame interventions must be co-developed with their target populations, potentially overlooking user concerns and preferences and impacting task relevance, enjoyment, and adherence (52). Additionally, more data on the feasibility, enjoyment, adherence, and effectiveness of the FitBrain program (or similar programs incorporating multiple dual-task and exergame approaches) should be collected in future studies to guide implementation better. The cost of various exergame and dual-task approaches should also be considered, identifying the cost-effectiveness of interventions that employ multiple technologies and interventions and comparing the effectiveness among several combinations of different technologies. Lastly, a more comprehensive and structured literature review should be conducted across various exergame and dual-task approaches, addressing issues with varied terminologies.

### 5.2 Research gaps and recommendations

Future research in dual-tasking and exergaming should focus on comprehensive feasibility studies and user-centered design to ensure safety, adherence, enjoyment, and relevance (114–118). Considering the variability of outcomes in dual-tasking and exergaming studies, incorporating standardized, sensitive, valid, and repeatable physical and cognitive measures is critical (119, 120). Studies should explore the combined effects of multiple dual-tasking and exergaming modalities. Additionally, future technologies and approaches in this field should aim to streamline workflows for more straightforward implementation in real-world settings (121–123). To the best of our knowledge, no study directly compares the effects of exergaming and dual-task training, and future research should consider designing studies to evaluate the comparative differences in outcomes between these two approaches. Lastly, more research should be conducted to evaluate the feasibility and effectiveness of programs combining multiple dual-tasking and exergaming approaches and comparing singular approaches to combined approaches in multiple populations.

## 6 Conclusion

Dual-tasking and exergaming offer significant health benefits, both physically and cognitively, across various clinical populations, particularly in older adults. These interventions, deliverable through diverse approaches and mediums, vary in cost and evidence base. Given their demonstrated benefits, integrating dual-tasking and exergaming into clinical, rehabilitation, and fitness settings warrants robust evaluation. Despite their growing popularity, it is crucial to focus on developing evidence-based interventions.

The brain gym program, exemplified by the FitBrain program, offers significant potential for improving cognitive and physical outcomes in older adults. Future research should focus on further validating this model through longitudinal studies and exploring its long-term impact on diverse populations. By integrating dual-tasking and exergaming into clinical practice, we can develop more effective, evidence-based interventions to support healthy aging.

## Data Availability

The original contributions presented in the study are included in the article/Supplementary Material, further inquiries can be directed to the corresponding author.
